# Popeye Domain Containing 1 (Popdc1/Bves) Is a Caveolae-Associated Protein Involved in Ischemia Tolerance

**DOI:** 10.1371/journal.pone.0071100

**Published:** 2013-09-16

**Authors:** Yifat Alcalay, Edith Hochhauser, Vitaly Kliminski, Julia Dick, Muayad A. Zahalka, Doris Parnes, Hadassa Schlesinger, Zaid Abassi, Asher Shainberg, Roland F. R. Schindler, Thomas Brand, Gania Kessler-Icekson

**Affiliations:** 1 The Felsenstein Medical Research Center, Sackler Faculty of Medicine, Tel-Aviv University, Tel-Aviv, Israel; 2 Department of Physiology, Rappaport Faculty of Medicine, Israel Institute of Technology, Haifa, Israel; 3 Faculty of Life Sciences, Bar-Ilan University, Ramat-Gan, Israel; 4 Harefield Heart Science Centre, Imperial College, London, United Kingdom; University of Central Florida, United States of America

## Abstract

Popeye domain containing1 (Popdc1), also named Bves, is an evolutionary conserved membrane protein. Despite its high expression level in the heart little is known about its membrane localization and cardiac functions. The study examined the hypothesis that Popdc1 might be associated with the caveolae and play a role in myocardial ischemia tolerance. To address these issues, we analyzed hearts and cardiomyocytes of wild type and Popdc1-null mice. Immunoconfocal microscopy revealed co-localization of Popdc1 with caveolin3 in the sarcolemma, intercalated discs and T-tubules and with costameric vinculin. Popdc1 was co-immunoprecipitated with caveolin3 from cardiomyocytes and from transfected COS7 cells and was co-sedimented with caveolin3 in equilibrium density gradients. Caveolae disruption by methyl-β-cyclodextrin or by ischemia/reperfusion (I/R) abolished the cellular co-localization of Popdc1 with caveolin3 and modified their density co-sedimentation. The caveolin3-rich fractions of Popdc1-null hearts redistributed to fractions of lower buoyant density. Electron microscopy showed a statistically significant 70% reduction in caveolae number and a 12% increase in the average diameter of the remaining caveolae in the mutant hearts. In accordance with these changes, Popdc1-null cardiomyocytes displayed impaired [Ca^+2^]_i_ transients, increased vulnerability to oxidative stress and no pharmacologic preconditioning. In addition, induction of I/R injury to Langendorff-perfused hearts indicated a significantly lower functional recovery in the mutant compared with wild type hearts while their infarct size was larger. No improvement in functional recovery was observed in Popdc1-null hearts following ischemic preconditioning. The results indicate that Popdc1 is a caveolae-associated protein important for the preservation of caveolae structural and functional integrity and for heart protection.

## Introduction

The Popeye domain containing (Popdc) family comprises three highly conserved, developmentally-regulated genes, *Popdc1-3*, expressed predominantly in muscles but also in epithelia and other cell types. *Popdc1*, also named *Bves* (blood vessel epicardial substance), is considered to be the founding member and being the most studied one, represents the prototype for the entire Popdc gene family [1]–[6].

Popdc1 possesses an extracellular N-glycosylated amino-terminus, three transmembrane domains and an intracellular carboxyl-terminus. The highly conserved Popeye domain, found in the intracellular segment, contains one or more homodimerization motifs and functions as cAMP binding domain [5], [7]–[9]. Studies in epithelial cells have indicated that Popdc1 plays a role in cell-cell interaction and adhesion and that the interaction of Popdc1 molecules with one another is important for the maintenance of intercellular junctions [8]–[10]. Involvement of Popdc1 in the regulation and signaling of tight junction formation and function, vesicular transport and receptor cycling has been demonstrated [11]–[14]. Hypermethylation of the *Popdc1* promoter was observed during tumorigenesis and was correlated with the downregulation of *Popdc1* expression [15]–[17] and mutated *Popdc1* has been identified in patients born with Fallot's tetralogy [18], suggesting a potential role for *Popdc1* in the control of cell growth and differentiation and in heart morphogenesis.

While *Popdc1* expression in muscles is several-fold higher than in epithelia [2], our knowledge of *Popdc1* function and regulation in the heart and skeletal muscles is rather poor. We identified a marked reduction in *Popdc1* expression in end stage failing human hearts [19]. In addition, *Popdc1*-null mice did not develop any obvious heart defect but displayed impaired skeletal muscle regeneration [3] and maladaption of heart rhythmicity to adrenergic stress that might be related to the ability of *Popdc1* and the other family members to bind cAMP and to interact with ion channels such as TREK-1 [20], [21].

Caveolae are cholesterol and glycosphingolipid-rich plasma membrane microdomains that contain the scaffolding protein caveolin and appear as 50–100 nm plasma membrane invaginations. The caveolae serve as dynamic docking sites to organize, traffic and regulate membrane and membrane-associated signaling complexes [22], [23]. The muscle specific caveolin3 (Cav3) and the caveolae have been found critical for cardioprotection and for ischemic preconditioning [24] and play a role in the modulation of calcium handling during excitation-contraction coupling and in hypertrophy [25]–[27]. Cav3 appears in the cardiomyocyte sarcolemma, intercalated discs and T-tubules and was identified in the costameres, rib-like perisarcolemmal multiprotein complexes that align with Z disks and T-tubules and function in cell adhesion, stretch-sensing and force transmission [28], [29]. Sequence analysis of Popdc1 revealed a putative caveolin binding motif within the highly conserved Popeye domain [30], suggesting Cav3 as a potential interacting protein and caveolae as a possible membrane site for Popdc1.

Given that Popdc1 is an abundant membrane protein in cardiac myocytes, we hypothesized that Popdc1 might reside in the caveolae and function in cardiac injury and protection. We report herein that Popdc1 is a caveolae-associated protein important for the maintenance of caveolae number and size. Accordingly, cardiomyocytes of the mutant hearts display impaired [Ca^+2^]_i_ transients, higher sensitivity to oxidative stress and no pharmacologic preconditioning while the mutant hearts are more vulnerable to I/R injury and show no ischemic preconditioning.

## Methods

### Animals

#### Ethics Statement

The study was carried out in strict accordance with the recommendations in the Guide for the Care and Use of Laboratory Animals of the National Institutes of Health. The protocol was approved by the Committee on the Ethics of Animal Care and Use of Tel-Aviv University (Permits number M- 05-068 and M-09-012). Surgery was performed under isoflurane anesthesia and all efforts were made to minimize suffering.

Wild type (WT) and *Popdc1*- null C57/BL/6J mouse colonies were established from the progeny of heterozygous (*Popdc1^+/−^*) breeders [31] and maintained in the Institutional Animal Facility under standard controlled conditions. Biometric information of the WT, *Popdc1-*null and heterozygotes is summarized in Table S1 in File S1. Unless otherwise stated, the measurements and experiments were performed on 3 month-old male mice. Rat neonates (Wistar, 24–48 h old) were purchased from Harlan Laboratories (Jerusalem, Israel).

### Isolated heart preparation and measurement of infarct size

Hearts were quickly removed from heparinized (500 U/kg), anesthetized (isoflurane inhalation) mice, cannulated through the aorta, and retrogradely perfused according to the Langendorff method using oxygenated, warmed (37°C) Krebs-Henseleit bicarbonate buffer (KHB) containing in mM: 118 NaCl, 2.4 KCl, 1.2 MgSO_4_×7H_2_O, 2. CaCl_2_, 5 EDTA, 1.2 KH_2_PO4, 25 NaHCO_3_, 4 glucose, and 2 pyruvate) at a constant perfusion pressure of 96 cm H_2_O as previously described [32]. To disrupt the caveolae hearts were perfused with KHB containing 0.2 mM methyl-beta-cyclodextrin (MβCD) for 20 min. The I/R protocol included 30 min of normoxic perfusion (stabilization), 30 min of global ischemia and 90 min reperfusion. For ischemia preconditioning (IPC), hearts underwent 20 min normoxic perfusion, 5 min no-flow ischemia and 10 min of reperfusion prior to the global ischemia. The pressure in the left ventricle (LV) was continuously recorded and the LV developed pressure (LVP, mmHg), the rates of pressure development and relaxation (dP/dt, -dP/dt, mmHg/min), the heart rate (HR, beats/min) and the rate·pressure product (RPP, beats/min·mmHg) were obtained. The coronary flow (CF) was assessed by measuring the coronary effluent drained from the pulmonary artery. Infarct size was assessed at the end of reperfusion using triphenyltetrazolium chloride (TTC) [33]. For further analyses, hearts were snap frozen in liquid nitrogen and stored at −70°C.

### Histology and electron microscopy

LacZ activity was visualized in 5 μm cryosections as previously described [31]. For confocal immune-histochemistry, paraformaldehyde-fixed cryosections were incubated with anti-Popdc1 (sc-49889), anti-Cav3 (sc-5310), anti-vinculin (sc-5573) and anti-connexin43 (sc-9059) primary antibodies (purchased from Santa Cruz Biotechnology) and Cy3-conjugated or DyLight 488-conjugated secondary antibodies (Jackson ImmunoResearch Laboratories). Nuclei were labeled by Hoechst 33342. For visualization and photography the Leica TCS SP5 Confocal Imaging System (Leica Microsystems, Germany) was employed using ×63 oil objectives. For electron microscopy, heart samples (1 mm^3^) were fixed in 2% glutaraldehyde, postfixed in 1% OsO_4_/0.1 M cacodylate buffer, dehydrated and embedded in Epon 812. Ultra thin sections (60 nm) were stained with 1% uranyl acetate and Reynolds's lead citrate and images were acquired using a Philips CM 12 ultramicroscope (at 60 kV) and a Morada CCD, iTEM camera (Olympus Soft Imaging Solutions, Münster, Germany). The number of caveolae per membrane length (in μm) and caveolae diameter (within the 50–100 nm diameter range) were measured in 250 randomly sampled images acquired at X40,000 magnification. A total of 680 and 660 µm membrane lengths were analyzed in WT and Popdc1/Popdc1-null hearts, respectively.

### Cell culture and transfection

Primary cultures of neonatal rat or mouse cardiomyocytes were prepared as previously described [34]. Rat cells were seeded (2×10^6^/ml) on collagen coated 100 mm culture plates and 72 h later proteins were extracted for co-immunoprecipitation (co-IP). Mouse cells were plated (5×10^5^/ml) on fibronectin coated glass cover slips and 72 h later the cells were processed for immuno-confocal microscopy using the above mentioned antibodies. COS7 cells were grown to 90% confluency on uncoated glass cover slips and co-transfected with expression vectors of full-length mouse Popdc1 and Cav3, using Lipofectamine2000 (Invitrogen, USA). At the end of 36 h incubation the cells were fixed and processed for immuno-confocal microscopy as above. For co-IP, Cos-7 cells were transiently transfected with murine Myc-tagged full-length Popdc1 and deletion mutant constructs Popdc1Δ92 or Popdc1Δ116 (Popdc1 lacking 92 or 116 amino acids at the C terminus) using Lipofectamine2000. Forty eight hours later, cells were lysed and processed for co-IP.

### Co-immunoprecipitation

(1) Neonatal rat cardiomyocytes were incubated with the membrane permeable crosslinker dithio-bis-succinimidylpropionate to stabilize protein complexes within the living cells. Proteins were extracted and incubated with either, rabbit anti-Popdc1 (sc-134807, Santa Cruz), mouse anti-Cav3 (sc-5310) antibodies or the corresponding IgG controls. Protein A-Sepharose beads were added and the Sepharose-bound immune complexes were spun down by centrifugation, eluted by boiling with Laemmli's sample buffer and subjected to WB analysis on 12% SDS-PAGE using sc-49889 and sc-5310 as primary antibodies for Popdc1 and Cav3 respectively. (2) Agarose bound anti-Myc antibody (ProFound c-Myc Tag IP/Co-IP Application Set, Fisher Scientific) was added to lysates of transfected COS7 cells. The bound proteins were eluted by boiling and subjected to WB analysis using mouse anti-Cav3 (BD Biosciences) as the primary antibody.

### Quantitative polymerase chain reaction (qPCR) and Western blot (WB) analysis

Total RNA was isolated from frozen hearts as previously described [19], cDNA was prepared and qPCR was performed using SYBR Green for product detection. The primer sequences used were: *Popdc1*, F: GCCTGCACCACTTTCTGC; R: CTCGATTGGCTTCATCTTGG. *Popdc2*, F: CTCAATGACAAGCTGTTTGCC; R: ATCTTTCTCAGACTCTGGTTCC.

*Popdc3*, F: CCTGAGTGGGATTCGCTAAG; R: CGGTGTCTGCTGTGAGAGTT.

*LacZ*, F: AACCCTGGCGTTACCCAACT; R: TCTTCGCTATTACGCCAGCT.

*Rps3*, F: AAGATGGCGGTGCAGATTTC; R: AGCCAGCTCCCGAGTGAGA. Expression values were obtained from C_t_ values detected by the StepOnePlus V2.1 software (Applied Biosystems, USA). The target gene levels were expressed as the N-fold difference in the target gene expression relative to RpS3 expression (ΔC_t_), where ΔC_t_ was determined in each sample by subtracting the average C_t_ value of the target gene from the average C_t_ value of the RpS3. Alterations in the levels of each mRNA during the experiment were calculated in Relative Quantity (RQ) values (calculated as 2^−ΔΔCt^) that were normalized further taking a randomly selected specimen of the “basal” group as 1. All mRNA scores are presented in arbitrary units. For WB, proteins extracted from frozen heart tissues (40 µg/lane) or sampled from sucrose gradient fractions, were resolved on 12% (5% for Ca_v_1.2) polyacrylamide-SDS gels, electroblotted to Hybond-C Extra membranes and incubated with the above listed goat anti-Popdc1, mouse anti Cav3, rabbit anti connexin43, rabbit anti Cav1.2a (acc-003, Alomone Labs, Israel), and mouse anti-actin (Clone C4, MP Biomedicals) primary antibodeis. Reactive bands were detected by AlexaFluor 680 Rabbit anti-goat, DyLight 800 goat anti rabbit or DyLight 800 goat anti-mouse secondary antibodies (Jackson Laboratories) as appropriate and quantified by the Odyssey™ Infrared Imaging System (Li-Cor Biotechnology, USA). A 10–170 kDa protein ladder (PageRuler™ Prestained Protein Ladder, Fermentas/Thermo Pierce) was present in every gel to estimate the molecular weights of reactive bands.

### Isolation of caveolin-rich fractions (caveolae)

Caveolin-enriched fractions were isolated as described by Kim et al. [35]. In brief, hearts were homogenized in 1% TritonX-100, 25 mM HEPES pH 6.5, 150 Mm NaCl, 1 mM EDTA, 1 mM Phenylmethylsulfonyl fluoride (PMSF), and protease inhibitor cocktail (Roche). Equal amounts of protein were brought to 40% sucrose, transferred to ultracentrifuge tubes and overlaid with 30% sucrose and 5% sucrose layers. Gradients were centrifuged at 28000 rpm and 4°C for 24 h and 12 equal fractions were collected from the top and sampled for WB analysis and cholesterol quantification (the AmplexRed kit, Invitrogen, USA). The Popdc1 reactive band was validated by mass spectrometry (The Smoler Proteomics Center, Technion, Haifa, Israel).

### Isolation of adult mouse cardiomyocytes and measurement of [Ca^2+^]_i_

Adult heart myocytes were isolated according to published protocols [36], [37] perfusing collagenase type II (Worthington Biochemical Corporation, USA) and hyaluronidase (Sigma, USA) as the dissociating enzymes in the presence of 12.5 µM CaCl_2_. The calcium concentration was gradually restored to 1 mM and the cells were taken for immediate use. Intracellular free calcium [Ca^2+^]_i_ was measured in individual cardiomyocytes using the indicator Indo-1-AM and a Zeiss epi-fluorescence inverted microscope as previously described [38], [39]. A fluorescence ratio of 410 nm/490 nm, which is proportional to [Ca^2+^]_i_, was acquired every 10 ms and the time integral of Ca^2+^ inux was calculated [38].

### Mitochondria permeability transition pore (mPTP) opening and pharmacological preconditioning (PPC)

Permeability of the inner mitochondrial membrane to the fluorescent dye calcein-AM indicates mPTP opening in intact cells [36], [40]. Adult heart cardiomyocytes were isolated as above with the addition of 10 mM 2,3-Butanedione monoxime (Sigma) to the perfusion buffer. To obtain PPC, cells were incubated with 1.5% isoflurane (Priamal Healthcare, India) in perfusion buffer for 30 min at 37°C. At the end of preconditioning, the cells were loaded with 1 µM calcein-AM (Invitrogen, USA) and 1 mM cobalt chloride (Sigma, USA) and distributed into 96-well FLUOTRAC 600 black polystyrene plates (USA Scientific, USA). Oxidative damage was induced by the addition of H_2_O_2_ (200 µM) and the decay of calcein fluorescence was monitored for 30 min at 480 nm excitation and 528 nm emission using the Synergy-HT Multidetection Microplate Reader (BioTek, USA).

### Statistics

Results are expressed as mean ± SEM unless otherwise indicated. Differences between experimental groups were evaluated for statistical significance using 2-tailed Student *t*- test for unpaired two group comparison. Response over time was analyzed by ANOVA for repeated measures. Probability value of *p*<0.05 was considered statistically significant.

## Results

### Popdc1 is a caveolae-associated protein

**Figure 1:**
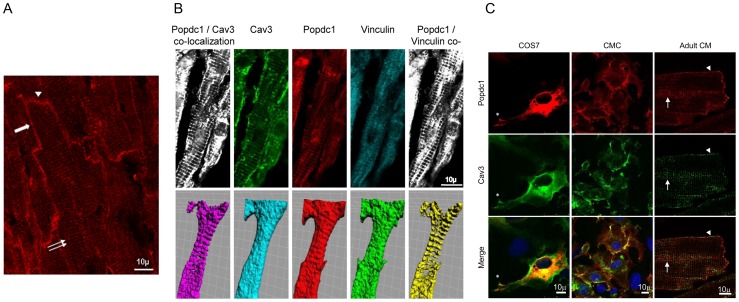
Localization of Popdc1 in WT cardiomyocytes. (a) Popdc1 (red labeling) is evident at the lateral membrane (arrow), intercalated discs (arrowhead) and transverse striations (double arrow). (b) Upper panel, co-labeling for Popdc1 (red), Cav3 (green) and vinculin (light blue); on the left, merge of Popdc1 with Cav3 (white) and on the right, merge of Popdc1 with vinculin (white). Lower panel, 3D surface analysis based on Z-stack pictures. Popdc1 (red), Cav3 (light blue), vinculin (green), merge of Popdc1 with Cav3 (violet), merge of Popdc1 with vinculin, yellow. Square size 5×5 µm. (c) Popdc1 (red) and Cav3 (green) co-localize (yellow) in the sarcolemma (arrowhead) and T-tubules (arrows) in isolated adult WT cardiomyocytes (Adult CM), at sites of cell-cell contacts in cultured neonatal WT cardiomyocytes (CMC), and in Popdc1/Cav3 co-transfected COS7 cells at sites of cell adhesion (Asterisk) and inside the cells.

We sought to identify Popdc1 within membrane domains that may indicate its role in the myocardium. Employing immunoconfocal microscopy we detected Popdc1 along the lateral sarcolemma and in the intercalated discs, as well as in transverse intracellular striations (Fig. 1a). Co-localization of Popdc1 with Cav3 suggested its association with sarcolemmal and T-tubular caveolae (Fig. 1b, c), while co-localization with vinculin implicated association of Popdc1 with the T-tubules and costameres (Fig. 1b) [41]. Lack of co-localization with myosin and alpha actinin excluded a structural relationship with the sarcomeres (not shown). Three dimensional surface analysis based on confocal Z-stack pictures confirmed the co-localization of Popdc1 with Cav3 and vinculin in periodic striations perpendicular to the long axis of the cell at approximately 2 µm intervals corresponding to the Z-disc alignment (Fig. 1b, lower panel). Co-localization with Cav3 was found also in cylindrical freshly isolated adult cardiomyocytes, in cultured neonatal cardiomyocytes and in COS7 cells co-transfected with Popdc1 and Cav3 expression vectors (Fig. 1c). In the cultured cardiomyocytes and COS7 cells, Popdc1 and Cav3 co-localization was found at sites of cell-cell contact, at points of cell adhesion and inside the cells but no striated organization could be seen (Fig. 1c).

**Figure 2:**
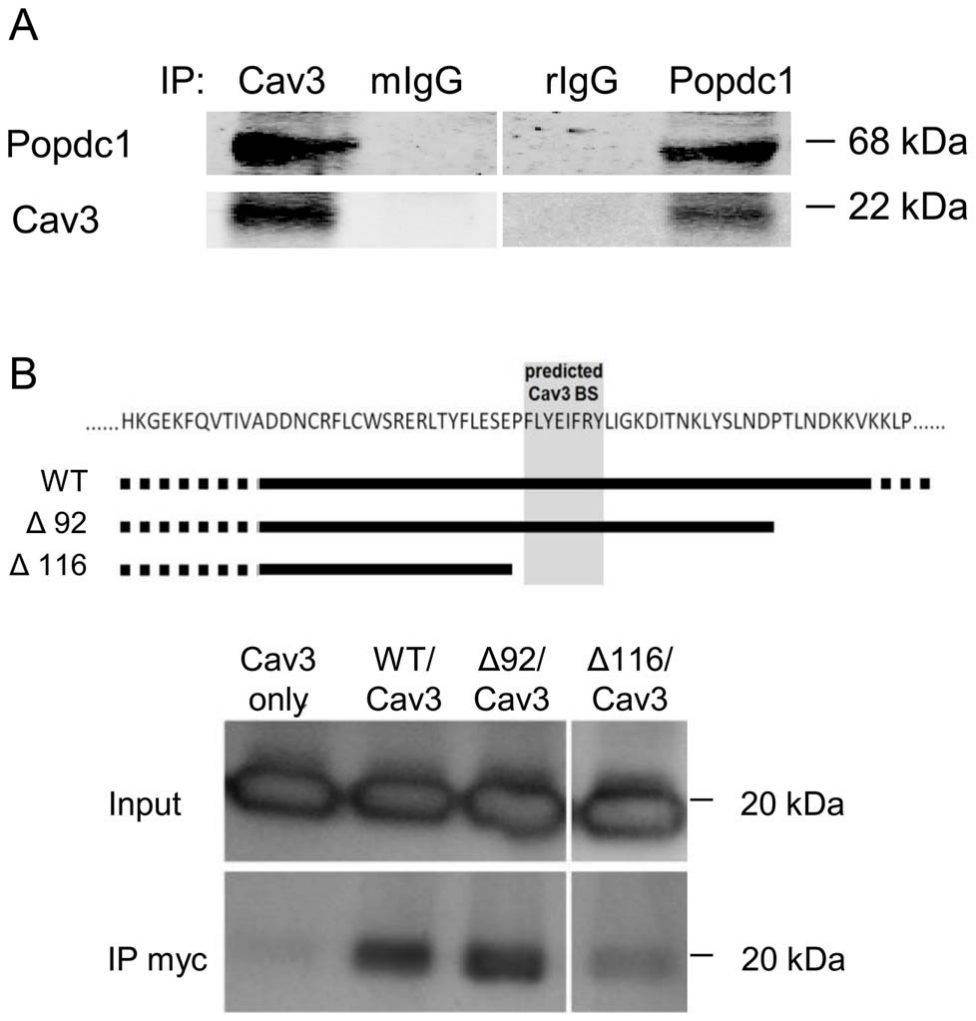
Co-immunoprecipitation of Popdc1 and Cav3. (a) Immunoblots demonstrating the precipitation of Popdc1 and Cav3 using mouse anti-Cav3 antibodies (left) and rabbit anti Popdc1 antibodies (right). Top labels: IP: immunoprecipitation tool; Cav3, anti-Cav3; mIgG, mouse immunoglobulins (control); Popdc1, anti-Popdc1 antibodies; rIgG, rabbit immunoglobulins (control). Left/right labels: Popdc1 migration at 68 kDa and Cav3 migration at 22 kDa. (b) Co-immunoprecipitation of Popdc1 and Cav3 from COS7 cells co-transfected with Cav3 and full length (WT) or deletion mutants (Δ92 and Δ116) Myc-tagged Popdc1 expression constructs. The scheme depicts the position of the deletions relative to the predicted Cav3 binding site (BS). Shown is a Western immunoblot probed for the detection of Cav3. Input, Cav3 in the original cell extract; IP-Myc, Cav3 that was precipitated by the anti-Myc antibodies. The figure depicts results of a representative membrane.

The overlap of Popdc1 and Cav3 observed in the confocal images suggested close localization and possible interaction of the two proteins. To test that, we attempted the co-immunoprecipitation of Popdc1 and Cav3 from cell extracts. As shown in Fig 2a, Popdc1 was precipitated from neonatal cardiomyocytes using antibodies against Cav3 while Cav3 was precipitated from the same cell extracts by antibodies against Popdc1. The co-immunoprecipitation indicated close association, if not direct interaction, of the two proteins in primary cardiomyocytes. Then, we examined the importance of the putative caveolin binding motif found within the Popeye domain for the interaction of Popdc1 with Cav3. We co-transfected COS7 cells with expression vectors for Cav3 and for Myc-tagged wild-type or deletion-mutated Popdc1 and looked for the immunoprecipitation of Cav3 by anti-Myc antibodies. Fig 2b demonstrates that wild type and deletion Δ92 Popdc1, which contained the putative caveolin binding motif, interacted with Cav3 in a manner that allowed the immunoprecipitation of Cav3 with antibodies specific for the Myc-tagged Popdc1. However, when the Popdc1 sequence lacked the putative caveolin binding motif (deletion Δ116), Cav3 was not co-precipitated, indicating that the caveolin binding motif is required for the interaction of Popdc1 with Cav3 and that the two proteins may interact directly with each other.

**Figure 3:**
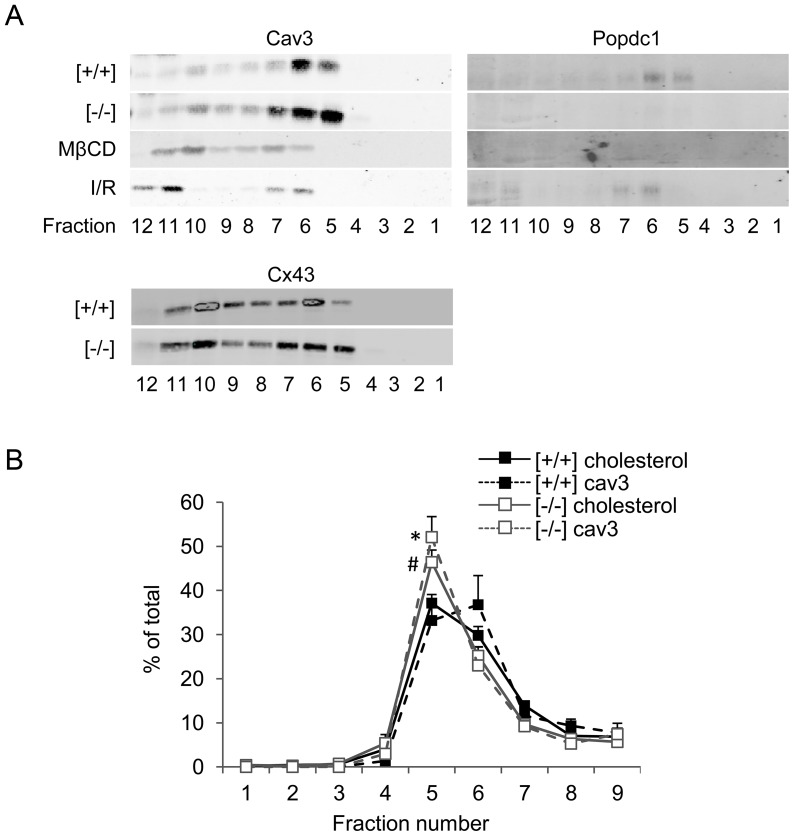
Popdc1 density sedimentation in discontinuous sucrose gradients. (a) Western blot analysis of gradient fractions. Fraction 1 and fraction 12 represent the lowest and highest density, respectively. [+/+], WT; [−/−], Popdc1-null; MβCD, methyl-β-cyclodextrin; I/R, ischemia/reperfusion; Cx43, connexin43. The molecular weights of the reactive bands were ∼68 kDa, ∼22 kDa, and ∼43 kDa, for Popdc1, Cav3, and Cx43, respectively. (b) The relative distribution of cholesterol and Cav3 throughout gradient fractions 1–9. *P<0.05, Cav3 in fraction 5, Popdc1-null vs. WT; ^#^P<0.05, cholesterol in fraction 5, Popdc1-null vs. WT.

**Figure 4:**
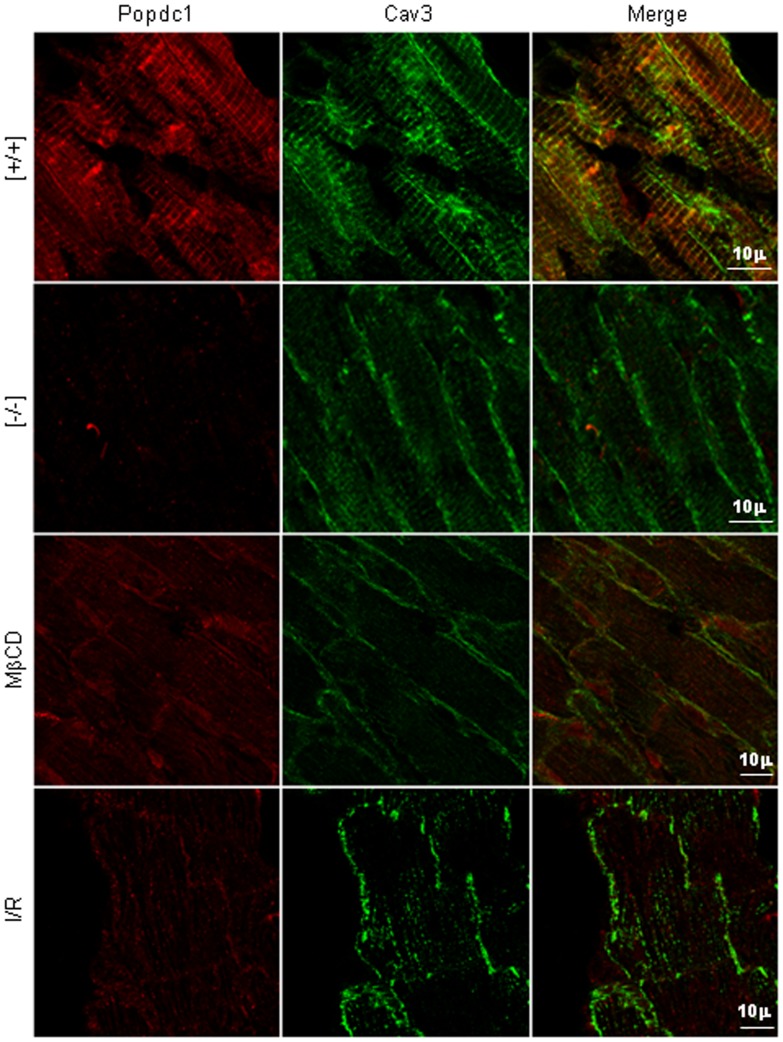
Popdc1 and Cav3 distribution in normal, mutant and injured hearts. Confocal microscopy images depicting Popdc1 (red) and Cav3 (green); co-localization sites appear in yellow. Note the low staining intensity of Cav3 cross striations in the *Popdc1*-null and MβCD-treated hearts, and the disappearance of Popdc1 and Cav3 co-localization following I/R.

The microscopic colocalization of Popdc1 with Cav3 and the ability to bind Cav3 pointed to the caveolae as a possible site for Popdc1. To examine that, we followed the sedimentation pattern of Popdc1 in density-gradient centrifugation of membrane preparations and found it within the Cav3-rich fractions (fractions 5 and 6) that represent the caveolae (Fig. 3a). Chemical disruption of the caveolae by cholesterol removal with MβCD redistributed Cav3 to fractions of higher density while Popdc1 distribution was below detection. Caveolae are dynamic structures susceptible to I/R injury [42]. In membrane preparations from I/R hearts undergoing 60 min reperfusion (see below) we found that the Cav3 rich fractions were redistributed to fractions 6–7 and 11–12 and that Popdc1 was shifted to the same fractions (Fig. 3a). The immunohistochemical analysis of cardiomyocytes in hearts subjected to MβCD infusion or the I/R protocol revealed no co-localization of Popdc1 with Cav3 (Fig. 4), suggesting that caveolae disruption by either chemical or physiological means altered the organization Popdc1 and Cav3 relative to each other.

**Figure 5:**
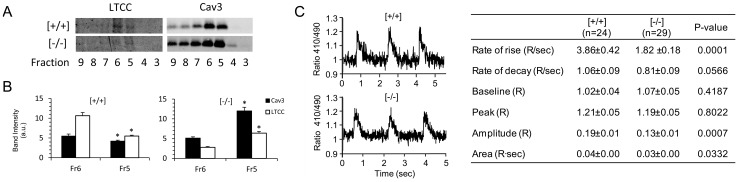
LTCC density sedimentation and [Ca^2+^]_i_ transients in Popdc1-null cardiomyocytes. (a) Representative WB depicting the sedimentation pattern of LTCC (Cav1.2a, ∼200 kDa) and Cav3 (∼22 kDa) in membrane preparations of WT [+/+] and *Popdc1*-null [−/−] hearts. (b) Quantification of the relative distribution of LTCC and Cav3 in gradient fractions 5 (Fr5) and 6 (Fr 6) of the two genotypes. The results summarize, in arbitrary units (a.u.), three and five independent experiments for LTCC and Cav3, respectively. *P<0.05, fraction 5 vs. fraction 6 within each genotype. (c) Left, [Ca^2+^]_i_ transients in representative cardiomyocytes; Right, summary of the measurements performed; n = number of cells; Mean ± SEM.

Density sedimentation of membrane preparations from *Popdc1*-null hearts revealed a shift of the Cav3-rich fractions towards a lower buoyant density, suggesting alterations in the caveolae profile in the mutant hearts (Fig. 3a). Other caveolae-associated proteins such as connexin43 or the L-type calcium channel were shifted, together with Cav3, to the lower density fractions (Fig. 3a, Fig 5). Calculation of the relative distribution of cholesterol and Cav3 throughout the sedimentation gradients demonstrated differences between the two genotypes (Fig. 3b). In WT hearts, cholesterol and Cav3 spanned fractions 5 and 6 while in Popdc1-null hearts both cholesterol and Cav3 peaked in fraction 5, reflecting reduced size-variation and lower buoyancy of the caveolae population in the null mutant hearts.

**Figure 6:**
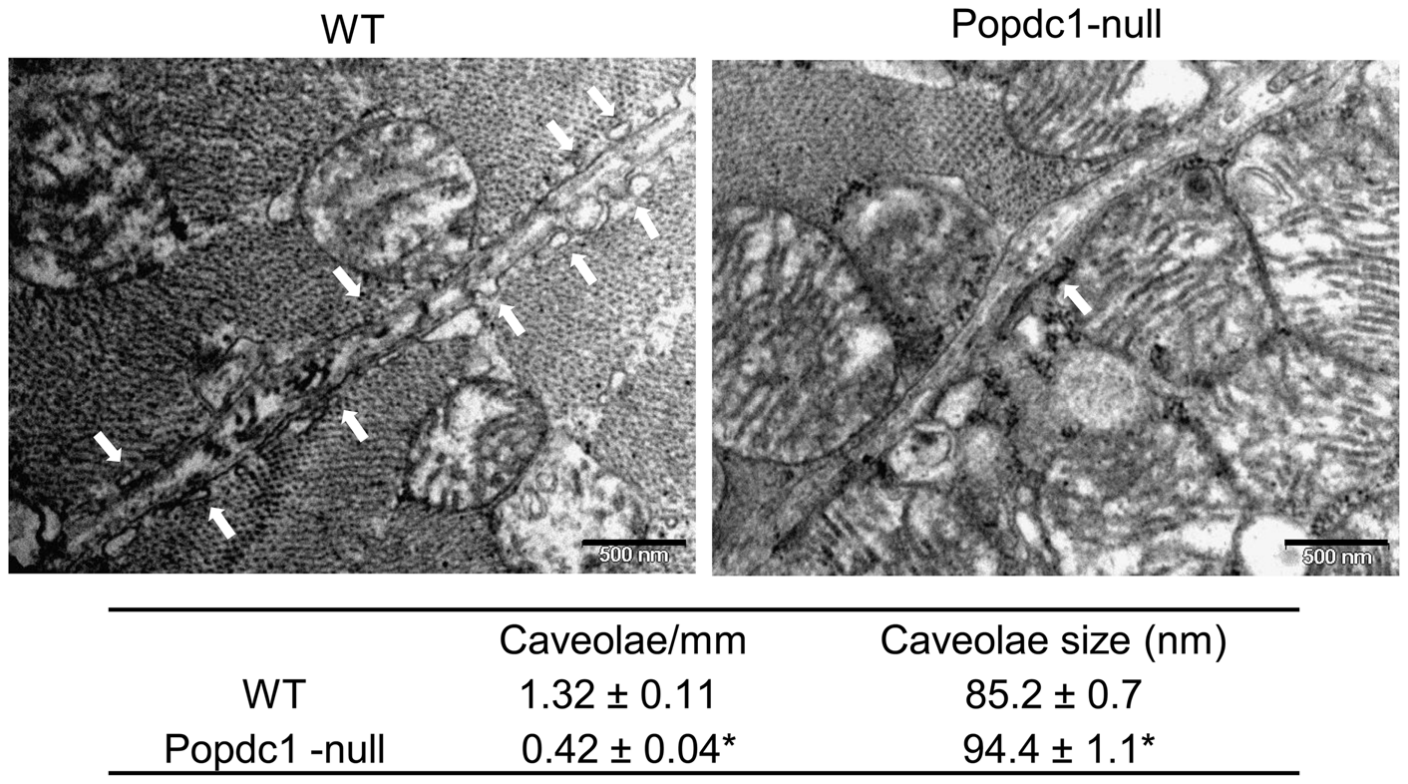
Caveolae are altered in *Popdc1*-null hearts. Electron micrographs showing caveolae (arrows) in WT and Popdc1-null hearts. Scale bar, 0.5 μm. In the bottom panel, summary of caveolae abundance and size measured along 680 and 660 mm membrane length in WT and Popdc1-null hearts, respectively; n = 3/genotype; Mean ± SEM; *P<0.00001.

The assessment of caveolae number and size by electron microscopy revealed a significantly lower abundance of caveolae per micrometer membrane-length (∼30%), and a significantly larger caveolae average size (∼12%), in the mutant compared with WT hearts (Fig. 6, P<0.00001 in each of the comparisons). It is suggested that Popdc1 plays a role in the maintenance of caveolae number and size. These alterations in the caveolae characteristics did not affect markedly the immunoconfocal presentation of Cav3, except for a small reduction in the labeling intensity of the t-tubular Cav3 (Fig. 4).

The results indicated that Popdc1 is a caveolae-associated protein involved in the maintenance of the distinctive density sedimentation of Cav3 and the proper caveolae number and size. It was, therefore, expected that cardiomyocytes and hearts lacking Popdc1 would display impairment of caveolae regulated functions. To test that we measured [Ca^2+^] transients in adult cardiomyocytes, LV functional recovery in isolated heart preparations, and the ability of hearts and cardiomyocytes to attain preconditioning.

### Impaired [Ca^2+^] transients in Popdc1-null cardiomyocytes

Caveolae disruption has been shown to impair [Ca^2+^]_i_ transients and Ca^2+^ sparks in cardiomyocytes [26], [27]. In the mutants, we detected a shift in the density sedimentation of the LTCC (Cav1.2a), which accompanied Cav3 in its sedimentation at a lower buoyancy (Fig. 5a, b), indicating its presence in the mutant caveolae. Therefore, we examined the importance of Popdc1 for calcium handling measuring the cytosolic Ca^2+^ in individual contracting cardiomyocytes. A significantly lower rates of rise (P = 0.00001) and of decay (p =  0.0566), and a smaller peak amplitude (P = 0.0007) of the [Ca^2+^]_i_ transients were measured in the *Popdc1*-null cells (Fig. 5c). This indicated reduced [Ca^2+^]_i_ transients in the absence of *Popdc1*, an expected outcome in the case of caveolae impairment [26].

### Impaired functional recovery in Popdc1-null hearts

**Figure 7:**
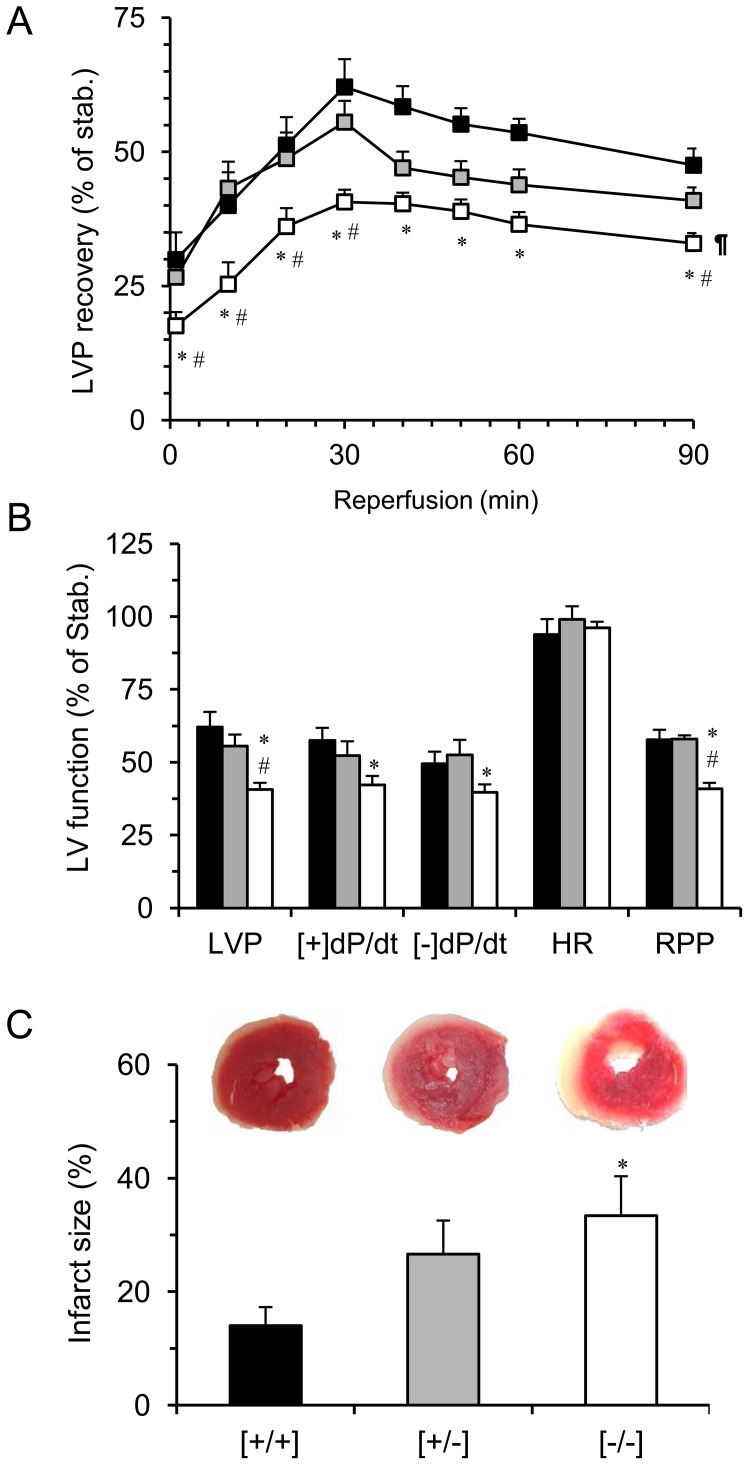
Heart function and infarct size. Hearts were subjected to Langendorff-perfusion as detailed in the Methods. (a) The LVP recovery during reperfusion starting at 1 min reperfusion. (b) Summary of LV function parameters at 30 min reperfusion. (c) Infarct size expressed as percent of the area at risk. *P<0.05, *Popdc1* ^[−/−]^ vs. *Popdc1* ^[+/+]^;^ #^P<0.05, *Popdc1* ^[−/−]^ vs. *Popdc1* ^[+/−]^. In the curve comparison (ANOVA with multiple repeats), ^¶^P<0.05, *Popdc1* ^[+/+]^ vs. *Popdc1* ^[−/−]^ and ^§^P<0.05. *Popdc1* ^[+/−]^ vs. *Popdc1* ^[−/−]^. Mean ± SEM; in (a) and (b) N = 12/group; in (c), N = 5/group.

We hypothesized that, in the mutants, the absence of *Popdc1* and the related caveolae impairment will increase the heart vulnerability to stressful conditions such as I/R injury and will abolish ischemic preconditioning (IPC). Hearts of WT and mutant heterozygotes and homozygotes were subjected to an I/R protocol of 30 min normoxic stabilization, 30 min global ischemia and 90 min reperfusion. No differences in LV function were detected between the three genotypes at the end of 30 min stabilization indicating normal basal cardiac performance in the absence of *Popdc1* (Table 1). Therefore, the hemodynamic parameters registered for each individual heart at this point were considered 100% performance. As shown in Figure 7, inferior functional recovery was found in the *Popdc1*-null compared to WT hearts following IR. The inferior recovery was evident from the first minute of reperfusion where the difference in the percent LVP recovery between mutant and WT hearts was ∼13% (P<0.05, Fig. 7a). The difference almost doubled as reperfusion advanced reaching 28% and 60% of LVP recovery at 30 min reperfusion that emphasized the importance of Popdc1 for the functional recovery. The same pattern of lower recovery was observed in dP/dt, -dP/dt, and RPP (P<0.05), whereas the heart rate (HR) was similar and stable in all the genotypes (Fig. 1b). The coronary flow did not vary between the three genotypes at any given time of I/R (data not shown), therefore, the differences in LV hemodynamics were not the outcome of impaired oxygen and nutrient supply and likely reflected intrinsic variation in the myocardial response to I/R. Of notice, the heterozygotes recovered similarly to WT hearts during the first 30 min of reperfusion; however, beyond that point, their LVP declined to values closer to those of the homozygote mutants (Fig. 7a). Corresponding to the functional analysis, a significantly greater infarct size was measured in hearts of Popdc1-null mice as compared to WT (P<0.05, Fig. 7c). The necrotic damage in hearts of heterozygotes was larger than in WT and smaller than in homozygotes, yet each of the comparisons did not reach statistical significance.

**Table 1:** Basal heart performance.

Genotype	LVP (mmHg)	+dP/dt (mmHg/sec)	−dP/dt (mmHg/sec)	HR (beats/min)	RPP (mmHg x beats/min)	CF (ml/min)
Popdc1^[+/+]^ (13)	86.5±9.2	2864±337	2164±274	337.5±15.3	29402±3560	2.6±0.2
Popdc1^[+/−]^ (18)	77.1±3.8	2345±152	1800±123	314.4±10.3	24370±1589	2.9±0.1
Popdc1^[−/−]^ (15)	84.5±6.2	2670±200	1971±151	345.7±16.6	29021±2327	2.8±0.1

Measurements taken at 30 min of normoxic Langendorff-perfusion (stabilization). LVP, left ventricular developed pressure; +dP/dt, rate of pressure development; −dP/dt, rate of pressure relaxation. HR, heart rate; RPP, rate⋅pressure product; CF, Coronary flow; Brackets, number of animals. Mean ± SEM.

**Figure 8:**
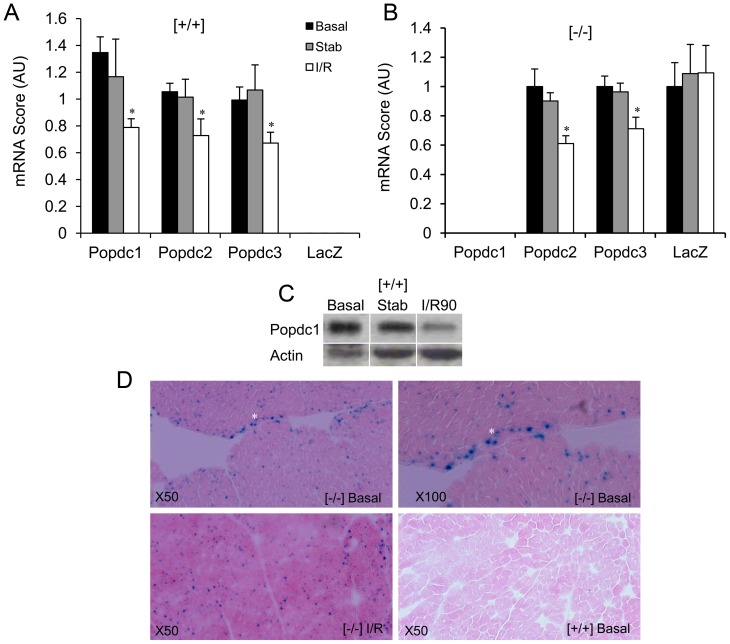
Popdc1 is down regulated by I/R. (a) and (b) RT-qPCR of Popdc1-3 and LacZ mRNAs from hearts isolated upon heart removal (Basal), at the end of stabilization (Stab) and at 90 min reperfusion (I/R). N = 5/group. AU, arbitrary units, Mean ± SEM; *P<0.05 compared to Basal. (c) Western blots of Popdc1 in WT hearts (Popdc1, ∼68 kDa; Actin, ∼42 kDa). (d) Cytochemical staining of LacZ activity (blue nuclei). Note, a higher intensity in subendocardial cells (white asterisk). Eosin counterstaining illustrates the general morphology. Images were captured at X50 and X100 magnification, as specified.

The expression level of *Popdc1* was significantly reduced in WT hearts following I/R while no effect was observed during the stabilization period (P<0.05, Fig. 8a, c). A significant and comparable reduction in transcript levels was measured for Popdc2 and Popdc3 mRNAs, both in the WT and the *Popdc1*-null mutant (P<0.05, Fig. 8a, b), indicating the susceptibility to I/R of all the three Popdc transcripts and no influence of *Popdc1* ablation on the regulation of the other family members (see also Table S2 in File S1). By contrast, the abundance of the LacZ mRNA remained unaffected by I/R (Fig. 8b) as was the distribution of nuclei positive for β-galactosidase (β-gal) activity (Fig. 8d). The cytochemical staining of cardiomyocyte nuclei for β-gal that reports on *Popdc1* expression was evenly distributed throughout the myocardium of the Popdc1-expressing mutants and higher staining intensity was observed in subendocardial cells that correspond to the conduction tissue (Fig. 8d).

### Popdc1 is essential for ischemic and pharmacologic preconditioning (IPC, PPC)

**Figure 9:**
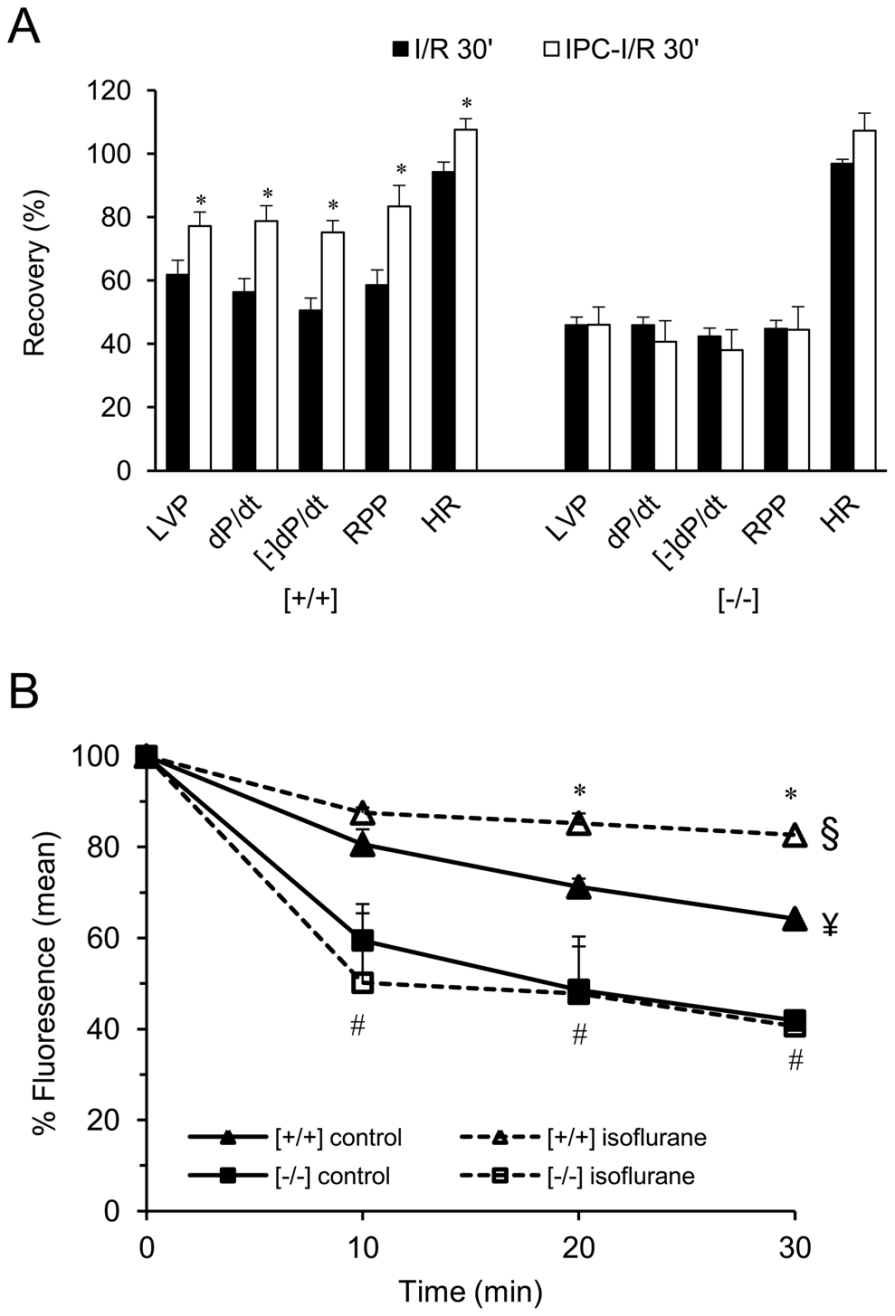
No preconditioning in *Popdc1*-null hearts and cardiomyocytes. (a) WT and mutant hearts underwent I/R perfusion with and without IPC. Parameters of LV function registered at 30 min reperfusion are shown. Labels are as in Figure 1. Mean ± SEM; *P<0.05, I/R vs. IPC-I/R within a genotype. (N = 5–9/group). (b) Wild type and mutant cardiomyocytes were preconditioned with isoflurane (1.5%) or left untreated, then loaded with calcein-AM and exposed to H_2_O_2_ (200 µM). The change in calcein fluorescence with time of H_2_O_2_ treatment is shown. Summary of three experiments performed in triplicates. *P<0.05, WT (*Popdc1* ^[+/+]^) cells, isoflurane vs. untreated control. ^#^P<0.05, control *Popdc1* ^[−/−]^ vs. control *Popdc1* ^[+/+]^ (isoflurane-untreated cells, open symbols). In the curve comparisons (ANOVA with multiple repeats), ^§^P<0.05, *Popdc1* ^[+/+]^ isoflurane vs. *Popdc* ^[+/+]^ control; ^¥^P<0.05 *Popdc1* ^[+/+]^ control vs. *Popdc1* ^[−/−]^ control.

Considering the fact that *Popdc1*-null hearts displayed inferior recovery from I/R and the available knowledge that no IPC and PPC can be obtained when caveolae are disrupted [43], [44], we hypothesized that *Popdc1*-null hearts and cardiomyocytes will display impaired preconditioning. To induce IPC, WT and mutant hearts were subjected to Langendorff perfusion whereby 5 min ischemia and 10 min normoxic perfusion preceded I/R injury and LV performance was recorded prior to and during reperfusion. Compared with controls, IPC significantly improved post-ischemic LV function in WT hearts (P<0.05) and no improvement was observed in the *Popdc1*-null hearts (Fig. 9a), indicating that Popdc1 is pivotal for IPC protection.

At the cellular level, the induction of oxidative injury in isolated cardiomyocytes, by H_2_O_2_, caused a significantly greater decay in calcein fluorescence in the mutant compared to WT cells (P<0.05) that indicated increased susceptibility for mPTP opening in cardiomyocytes lacking Popdc1 (Fig. 9b). Furthermore, the induction of PPC by the addition of isoflurane to the isolated cardiomyocytes attenuated the decay in calcein fluorescence (mPTP opening) in the WT (P<0.05) but not in the mutant cells that were not protected against the H_2_O_2_ injury (Fig. 9b). We conclude that similar to the whole hearts, the *Popdc1*-null cardiomyocytes displayed higher vulnerability to oxidative stress and failed to activate preconditioning.

## Discussion

Whereas Popdc1 (Bves) has been extensively studied in normal and transformed epithelial cells of various origins [4]–[6], [8]–[12], [15], [17], [19], there have been fewer reports regarding the function of Popdc1 in muscles although these tissues are the predominant site of *Popdc1* expression. In this work, we demonstrate that Popdc1 is a caveolae-associated protein essential for the maintenance of caveolae number and size and that *Popdc1* deficiency impairs Ca^2+^ handling, reduces the tolerance of I/R and oxidative stress, and abolishes ischemic and pharmacologic preconditioning.

### Popdc1 is a caveolae-associated protein

The caveolae serve as a dynamic structural platform that organizes and modulates the activity of distinct ion channels, receptors and signaling molecules through their interaction with caveolin [24], [45]. In the heart, the caveolae were shown to regulate Ca^2+^ homeostasis, ischemia tolerance, hypertrophy, stretch response and more [46]. While I/R injury reduced caveolae abundance and IPC increased their number [47], caveolae disruption abolished ischemic or pharmacologic preconditioning [48], [49] and Cav3 overexpression mimicked IPC by increasing caveolae number and improving I/R resistance [47].

Our conclusion that Popdc1 is a caveolae-associated protein is drawn from several observations that link it with Cav3, the caveolae scaffold protein: (a) Popdc1 co-localized with Cav3 in immuno-stained tissues and cells, (b) Popdc1 co-sedimented with Cav3 in equilibrium density gradients, and (c) Popdc1 co-immunoprecipitated with Cav3 from primary cardiomyocytes and from a transfected fibroblast-like cell line, COS7. While the precipitation of Popdc1 from cardiomyocytes by anti Cav3 antibodies could be indirect through Popdc1 binding to an intermediary protein within the Cav3 complex with no direct binding to Cav3, evidence from the transfected COS7 cells strongly supports a direct physical interaction of Popdc1 with Cav3. This view is mainly based on the fact COS7 cells only express Popdc1 and Cav3 when they were co-transfected with the corresponding coding sequences. Moreover the spontaneous interaction of the two proteins was lost when the putative caveolin-binding motif at the Popdc1 C-terminus was deleted. The latter indicates clearly that the Cav3 target sequence in Popdc1 is functional and necessary for the binding of Podpc1 to Cav3 and strongly supports a direct interaction of the two proteins.

In intact heart tissues, the appearance of Popdc1 in intracellular transversal striations and its co-localization there with Cav3 and vinculin, indicated presence in the T-tubules and the costameres. In cardiomyocytes, each of these structures has been shown to contain Cav3 and vinculin, and align with the sarcomeric Z discs [28], [41]. While freshly isolated adult ventricular cardiomyocytes that possess T-tubules display Popdc1 in transversal striations, neonatal cardiomyocytes and COS7 cells that lack T-tubules, do not. Previous studies that reported immunohistochemical labeling of Popdc1 in the heart did not show the “ribbed” staining either because the tissues were not of sufficient maturity or else, the antibodies used were not adequate or the microscopy was not optimal in terms of magnification, resolution or tissue preparation.

The co-localization of Popdc1 and Cav3 disappeared when caveolae were disrupted chemically (MβCD) or physiologically (I/R), suggesting an alteration in their alignment relative to each other. In the null-mutants, the distribution of Cav3 and other caveolae proteins to a lower buoyant density pointed to caveolae modification in these cells. The importance of Popdc1 for caveolae integrity was emphasized by a striking reduction in caveolae number and a larger average size of the remaining caveolae (measured within the consensus 50–100 nm size range) in the hearts lacking *Popdc1*. Whether this caveolae population represents aberrant caveolae or a normally existing subtype of Popdc1-free caveolae is as yet unclear. Nonetheless, the distinct *Podpc1*-deficient caveolae seem to be sufficient to sustain the heart as long as it is unstressed. Reports on caveolae classification according to size and function are scarce. Three caveolae subclasses have been characterized in adipocytes that differed in density, cholesterol content, and distinct subsets of specific proteins engaged in different functions in the plasma membrane such as fatty acid uptake or cholesterol metabolism [50]. Caveolae sub-populations that segregate according to their sarcolemma or T-tubule location and their occupant modulators of Ca^2+^ signaling to the sarcoplasmic reticulum have been described in the heart [51]. The larger, Popdc1-deficient caveolae may differ in their functional capabilities due to variations in the amount and repertoire of the proteins clustered by them. The lower abundance of these caveolae suggests a decrease in caveolae formation and/or stability.

Popdc1 contains two cholesterol interaction/recognition amino acid consensus sequences (CRAC), at the boundary of the 3^rd^ transmembrane domain and the cytoplasmic region (LSYLLYKKRPVK, a.a. 108–119), and at the top of the Popeye domain (LGGVYHR, a.a. 124–130), as well as a caveolin binding motif (FLYEIFRY, a.a. 244–251) located within the Popeye domain. The CRAC motif is present in caveolins and in proteins targeted to lipid rafts or regulating cholesterol metabolism and transport [52]. The caveolin binding motif is found in proteins that interact with the caveolin scaffolding domain [45]. Together, these motifs point to the potential involvement of Popdc1 in cholesterol recruitment and in caveolae formation or stabilization. It has been recently questioned whether a caveolin-binding motif, which consists of a number of aromatic amino acid residues is indeed functionally implicated into caveolin interaction since in most proteins this motif was found to be non-accessible [53]. Our own structural analysis of the location of the caveolin-binding motif in Popdc1 suggests that the caveolin binding site is part of an alpha-helix at the end of the Popeye domain and appears to be fully accessible since it is predicted to be on the protein surface.

### Popdc1 maintains caveolae-dependent functions

Much experimental evidence has established the importance of intact caveolae population for different cellular functions within the cardiomyocyte [22], [24], [44], [45], [47]–[49]. In this report, three known caveolae-dependent functions, including calcium homeostasis during contraction, the recovery from I/R injury and the capability of preconditioning were impaired or lost in hearts and cardiomyocytes lacking Popdc1, supporting the notion that Popdc1 acts to maintain caveolae-dependent functions. [Ca^2+^]_i_ cycling during contraction is regulated by the voltage dependent L-type Ca^2+^ channels and the Na^+^/Ca^2+^ exchanger, both are present in the caveolae, thereby facilitating Ca^2+^ signaling to the sarcoplasmic reticulum and Ca^2+^ extrusion through the sarcolemma [27], [54], [55]. Our findings of reduced [Ca^2+^]_i_ transient amplitude and slower rates of [Ca^2+^]_i_ rise and decay in Popdc1 deficient cardiomyocytes are compatible with the changes in [Ca^2+^]_i_ transients and Ca^2+^ sparks reported in MβCD treated cardiomyocytes [26], [27]. In the mutants, the shift of the L-type calcium channel, together with Cav3, to fractions of lower buoyancy suggested its distribution with the distinct Popdc1-deficient caveolae that might eventually affect the Ca^2+^ fluxes.

Whereas under basal conditions the *Popdc1*-null hearts displayed normal LV function, the importance of this highly conserved gene became visible under the stress of I/R when compensatory mechanisms failed to maintain the normal functional capacities. Perturbation of [Ca^2+^]_i_ cycling might be responsible for the depressed left ventricular contractility (±dP/dt) of the *Popdc1* deficient hearts following I/R [56]. The inferior functional recovery and greater infarct size in the *Popdc1*-null hearts provided clear evidence that this protein plays a role in the response to ischemia or in the recovery process. In the same line, the experiments in adult cardiomyocytes demonstrated that *Popdc1* deficiency increases the susceptibility to oxidative stress since cell exposure to H_2_O_2_ induced a greater damage to the Popdc1-null compared with WT cardiomyocytes.

Regarding the heterozygote I/R hearts, impaired recovery was detectable only after 30 min of reperfusion when the LV performance dropped from WT levels to nearly the null mutant levels as if some resources, structural or others, were depleted. This observation and the intermediate infarct size – between WT and the null mutants – suggest that the degree of functional impairment corresponds to the expression level of *Popdc1*. Namely, a partial deficit due to haploinsufficiency is enough to unmask the importance of Popdc1 in the response to I/R.

In the WT hearts, the levels of Popdc1 and its mRNA were both decreased by I/R. This could be the outcome of the acute tissue injury but could also contribute to the concomitant reduction in LV performance. Previously, we reported the diminution of Popdc1 and its coding mRNA in end stage heart failure in humans that suggested a relationship between the evolution of myocardial dysfunction and the decrease in Popdc1 [19]. Interestingly, the expression level of LacZ (the product of the Popdc1LacZ allele) was not affected by I/R. Since the *Popdc1*-null allele was generated by knock-in of a nuclear-targeted LacZ into the first coding exon of *Popdc1* [31], the originating transcript lacks the 3′-UTR and may resist mRNA destabilizing signals that target the 3′-UTR [57]. Besides, the nuclear localization of β-gal may rescue it from processes that degrade Popdc1.

The third indication that Popdc1 is important for functions governed by the caveolae was the inability of *Popdc1*-null hearts to undergo ischemic preconditioning and the failure of Popdc1-null cardiomyocytes to attain pharmacologic preconditioning. Previous studies have shown that caveolae disruption by chemical, physiological or genetic manipulations abolished preconditioning and deregulated proteins in the Cav3 complex that control cytoprotective pathways [42], [43], [49], [58]. These proteins, including signaling complexes that traffic from the sarcolemma to the mitochondria [49], ion channels that modulate protection signaling [58] and pro- and anti-survival MAP kinases [42], [43], were not investigated in this study. However, it is conceivable that many of them would be deregulated in the *Popdc1*-null hearts primarily because the caveolae are altered. The mechanism by which Popdc1 affects the caveolae population is still to be elucidated.

We relate much of the functional deficits of the Popdc1-null hearts to their impaired caveolae population however, part of the changes observed may evolve from the role of Popdc1 in other cellular or membrane domains that have not yet been elucidated. It has been recently shown that Popdc1 (Bves) facilitates vesicular transport in epithelial cells [13]. Participation of Popdc1 in vesicular transport in cardiomyocytes is possible and may or may not involve the caveolae. Popdc1 may also participate in cellular signaling through its recently demonstrated ability to bind cAMP and to interact with ion channels such as TREK-1 [20], [21]. TREK-1 has been shown to be cytoprotective after ischemic insults in the brain [59], however at present it is unclear whether TREK-1 has a similar role in the heart. Likewise it is at present unclear to what extent the ability of Popdc proteins to bind cAMP and to act as a signal mediator is important in the context of Cav3 binding and cardiac ischemia and preconditioning. In addition, as caveolae play a role in skeletal muscle differentiation and myoblast fusion [60], our findings may explain, at least in part, the retardation in muscle regeneration in *Popdc1*-null mice [31].

Two main issues were addressed in this study, the identification of sites where Popdc1 resides in the sarcolemma, and the investigation of Popdc1 importance for myocardial protection. The results demonstrated that Popdc1 is a caveolae-associated protein involved in caveolae maintenance and pointed out the importance of Popdc1 for several functions that depend on intact caveolae, including protection. A detailed mechanistic explanation has not been delineated in this study. Questions including the structural or functional association of Popdc1 with different proteins in the Cav3 complex, the involvement of Popdc1 in caveolae formation or in the mobilization of proteins to or from the caveolae have not been addressed and await future investigation.

### Conclusions

Our data provide the first evidence to date that Popdc1 is a protein of the caveolae and a major player maintaining their structural and functional integrity. Ablation of *Popdc1* exacerbates myocardial ischemic and oxidative injury and abolishes preconditioning protection. These observations indicate a role for Popdc1 in heart malfunction and disease pointing to caveolae mediated mechanisms.

## Supporting Information

File S1 (DOCX)
