# Supplementary material for: Popeye Domain Containing 1 (Popdc1/Bves) Is a Caveolae-Associated Protein Involved in Ischemia Tolerance
Source: PLoS One. 2013 Sep 16;8(9):e71100. doi: 10.1371/journal.pone.0071100 (PMC3774711; doi:10.1371/journal.pone.0071100)
Supplement: File S1 — (DOCX) [file pone.0071100.s001.docx]

**Popeye domain containing 1 (Popdc1/Bves) is a caveolae-associated protein involved in ischemia tolerance**

Alcalay Yifat^a^, Hochhauser Edith^a^, Kliminski Vitaly^a^, Dick Julia^a^, Zahalka A Muayad^a^, Parnes Doris^a^, Schlesinger Hadassa^a^, Abassi Zaid^b^, Shainberg Asher^c^, Schindler Roland^d^, Brand Thomas^d^, Kessler-Icekson Gania^a§^

^a^The Felsenstein Medical Research Center, Sackler Faculty of Medicine, Tel-Aviv University, Tel-Aviv, Israel, ^b^Department of Physiology, Rappaport Faculty of Medicine, Israel Institute of Technology, Haifa, Israel, ^c^Bar-Ilan University, Ramat-Gan, Israel and ^d^Harefield Heart Science Centre, Imperial College, London, United Kingdom

**Supportive information on the Popdc1-null mouse phenotype**

The Popdc1-null mouse was generated by NLS-LacZ knockin into the first coding exon of Popdc1 [1].

While earlier observations have suggested no overt phenotypic alterations in the Popdc1-null mutant [1], biometric data collected by us in 3 mo old male mice showed lower body and heart weights of Popdc1^[-/-]^  compared to Popdc1^[+/-]^ and Popdc1^[+/+]^ mice. However, the ratio of heart weight-to-body weight was similar in the three genotypes (Table ST1), indicating a slower gain in muscle mass and comparable restriction of heart growth that maintained the normal ratio of heart weight-to-body weight. Lower muscle mass and normal heart weight-to-body weight ratios were reported in several other mouse models including the Popdc2-null mutant [2]. In addition, we measured a higher systolic blood pressure in the Popdc1-null mutant. As the blood pressure values were within the published range (80-120 mmHg) for C57/BL-6J mice, the Popdc1-null mutant may be considered not hypertensive [3-5]. Nonetheless, the underlying cause for the observed discrepancy in blood pressure warrants investigation.

Table ST1: Biometric data

| Genotype | [+/+] | [+/-] | [-/-] |
| --- | --- | --- | --- |
| HW (mg) | 160±20 (13) | 165.2±10 (18) | 134.1±20 ^#^ * (15) |
| BW (g) | 29.75±2.25 (13) | 30.22±3.01 (18) | 24.65±2.51^#^ * (15) |
| HW/BW (%) | 0.54±0.05 (13) | 0.6±0.03 (18) | 0.54±0.04 (15) |
| BP (mmHg) | 90.4±11.3 (11) | 81.3±6.8 (5) | 119.9±24.8** (7) |
| HR (beats/min) | 390±33.5 (11) | 372.3±34.4 (5) | 401.2±36.6 (7) |

Three month-old male mice; HW, heart weight; BW, body weight; HW/BW, the ratio of HW to BW; BP, systolic blood pressure Mean±S.D., ^*^P<0.05 Popdc1^[-/-]^ vs. Popdc1^[+/+]^; ^#^P<0.05 Popdc1^[-/-]^  vs. Popdc1^[+/-]^. **P< 0.02, Popdc1^[-/-]^  vs. Popdc1^[+/+]^ and Popdc1^[+/-]^. In brackets, number of animals

Assessment of Popdc1-3 and LacZ transcripts revealed that the abundance of Popdc2 mRNA was ca. 16-fold higher, and that of Popdc3 was ca. 2.5-fold lower than Popdc1 in WT hearts, and that Popdc1-null mutants maintained normal levels of Popdc2 and -3 transcripts (Table ST2). The abundance of LacZ mRNA was 7-fold lower than expected from the Popdc1 expression in WT hearts. In the heterozygotes, the expression levels of Popdc1 and LacZ were nearly half their levels in the respective homozygotes, Popdc1^+/+^ and opdc1^-/-^LacZ^+/+^.

Table ST2: Popdc1-3 and LacZ mRNA relative expression in the three genotypes

RT-qPCR. Arbitrary units; n=5; *P<0.05 vs. each homozygote.

**References**

1. Andree B, Fleige A, Arnold HH, Brand T (2002) Mouse Pop1 Is Required for Muscle Regeneration in Adult Skeletal Muscle. Mol Cell Biol 22: 1504-1512.

2. Froese A, Breher SS, Waldeyer C, Schindler RF, Nikolaev VO, et al. (2012) Popeye domain containing proteins are essential for stress-mediated modulation of cardiac pacemaking in mice. J Clin Invest 122: 1119-1130.

3. Faury G, Pezet M, Knutsen RH, Boyle WA, Heximer SP, et al. (2003) Developmental adaptation of the mouse cardiovascular system to elastin haploinsufficiency. J Clin Invest 112: 1419-1428.

4. Gonzalez-Villalobos RA, Seth DM, Satou R, Horton H, Ohashi N, et al. (2008) Intrarenal angiotensin II and angiotensinogen augmentation in chronic angiotensin II-infused mice. Am J Physiol-Ren Physiol 295: F772-F779.

5. van den Borne SWM, van de Schans VAM, Strzelecka AE, Vervoort-Peters HTM, Lijnen PM, et al. (2009) Mouse strain determines the outcome of wound healing after myocardial infarction. Cardiovasc Res 84: 273-282.
